# Microphthalmia and microcornea: In congenital cytomegalovirus

**DOI:** 10.4103/0301-4738.53065

**Published:** 2009

**Authors:** Kava Maina P, Nagarajan Lakshmi

**Affiliations:** Department of Neurology, Princess Margaret Hospital, Roberts Road, Subiaco, Western Australia

Dear Editor,

Cytomegalovirus (CMV) infection is one of the most common viral causes of congenital infections and a contributor to neurodevelopmental disabilities in children. Microphthalmia and microcornea are rarely described in congenital CMV infection.

A 30-month-old girl presented at six months of age for microcephaly and developmental delay. She had generalized hypotonia, severe developmental delay, severe visual impairment and normal hearing. Her ophthalmologic assessment revealed bilateral cataracts, milky cornea, microphthalmia, posterior lenticular plaques. Her cornea measured 6mm horizontally and 7mm vertically in both eyes.

A computed axial tomography scan and magnetic resonance imaging (MRI) of the brain revealed calcifications in the subependymal region, basal ganglia, periventricular white matter and cerebellum. There was also reduced white matter bulk in the parieto-occipital region, delayed myelination, and hypoplastic optic nerves (on orbital MRI).

A B-scan ultrasound of the eyeballs revealed eyeball size of 18 mm in anterior posterior diameter and 20mm in transverse diameter and persistent hypertrophic vitreous with few small foci of echogenicity within the vitreous.

She had normal female karyotype (46XX), a normal urine metabolic screen (including urine amino acids, organic acids and mucopolysaccarides). Results for syphilis (Treponema pallidum antibodies not detected), rubella (serology = IgG < 5.0 IU/ml, IgM negative), herpes (Herpes Simplex serology Type 1 IgG, Type 2 IgG and IgM were negative) and other common viruses were non-conclusive. Toxoplasmosis was an important differential diagnosis, however, the toxoplasma IgG was 0.0 IU/mL (<2 IU/mL is negative). The CMV serology at six months of age was positive with an IgG = 164.8 AU/ml (Antibody Units per ml - Abbott definition; > = 15 AU/ml is positive) and a negative IgM. Urine CMV at six months was negative but was positive at 30 months. The cerebrospinal fluid (CSF) did not reveal any cells and the CSF, CMV, DNA as well as the CMV culture was negative. The CSF also did not reveal any lymphocytosis, elevated protein or tachizoites which are seen with congenital toxoplasmosis.The plasma CMV viral load (copies/ml) was less than 6.00 × 10^2^.

Ophthalmic manifestations of congenital CMV infection include chorioretinitis with secondary pigmentary retinitis and macular scarring, optic atrophy and central cortical defects in the lens.[1] Optic nerve hypoplasia, optic nerve coloboma, corneal opacities and bilateral anterior polar cataracts have been described in association with cytomegalic inclusion disease.[2] Uniocular micropthalmia with cataract has been reported in a neonate who had congenital CMV liver fibrosis.[3]

Microphthalmos is an eye that has an axial length less than 21 mm in an adult or less than 19 mm in a one-year-old child.[4] An adult cornea less than 10 mm in horizontal diameter is called microcornea. Microcornea may occur as part of microphthalmos. Most cases are sporadic although autosomal recessive and autosomal dominant pedigrees are reported.[5] The child had microcornea out of proportion to the microphthalmos.

Microphthalmia and microcornea are not abnormalities characteristically associated with congenital CMV infection. When seen in the context of a possible intrauterine infection, alternative diagnoses such as congenital rubella or toxoplasmosis or metabolic disorders should be sought.

It is generally accepted that a diagnosis of congenital CMV infection is established if the infant presents with clinical symptoms compatible with CMV disease in the first three weeks of life and has confirmatory laboratory evidence. However, the child first presented at six months of age. She had microcephaly, developmental disability, severe visual impairment, ophthalmologic and neuroimaging abnormalities and laboratory evidence of CMV infection. She fits within the category of “possible congenital CMV disease” described as CMV disease identified on the basis of clinical symptoms and confirmation of infection between four weeks and 12 months of age.[6]

In the absence of a family history of eye abnormalities, the microphthalmos and microcornea in our case is almost certainly secondary to congenital CMV infection.
